# A graph-based approach for simultaneous semantic and instance segmentation of plant 3D point clouds

**DOI:** 10.3389/fpls.2022.1012669

**Published:** 2022-11-10

**Authors:** Katia Mirande, Christophe Godin, Marie Tisserand, Julie Charlaix, Fabrice Besnard, Franck Hétroy-Wheeler

**Affiliations:** ^1^ Laboratoire Reproduction et Développement des Plantes, Univ Lyon, ENS de Lyon, UCB Lyon 1, CNRS, INRAE, Inria, Lyon, France; ^2^ Laboratoire ICube, University of Strasbourg, Strasbourg, France

**Keywords:** instance segmentation, semantic segmentation, Fiedler vector, quotient graph, phenotyping

## Abstract

Accurate simultaneous semantic and instance segmentation of a plant 3D point cloud is critical for automatic plant phenotyping. Classically, each organ of the plant is detected based on the local geometry of the point cloud, but the consistency of the global structure of the plant is rarely assessed. We propose a two-level, graph-based approach for the automatic, fast and accurate segmentation of a plant into each of its organs with structural guarantees. We compute local geometric and spectral features on a neighbourhood graph of the points to distinguish between linear organs (main stem, branches, petioles) and two-dimensional ones (leaf blades) and even 3-dimensional ones (apices). Then a quotient graph connecting each detected macroscopic organ to its neighbors is used both to refine the labelling of the organs and to check the overall consistency of the segmentation. A refinement loop allows to correct segmentation defects. The method is assessed on both synthetic and real 3D point-cloud data sets of *Chenopodium album* (wild spinach) and *Solanum lycopersicum* (tomato plant).

## 1 Introduction

3D acquisition systems of plants and crops have become widespread in recent years. This has allowed for automatic and non-destructive measurement of traits directly on the output of these systems, enabling automatized computational phenotyping. In the case of complex traits (leaf areas, stem length, internode distances, etc.), this is however only possible when plant organs are automatically identified, as explained in the review made by (Paulus, 2019). This means two things. First, the virtual representation of the plant, usually a 3D point cloud, should be decomposed into parts corresponding to the plant organs. Second, each part should be labelled according to the organ it represents. The first task is often called *instance segmentation*, or sometimes *part segmentation*. The second one is known as *semantic segmentation* or *classification*.

Instance segmentation is often based solely on the local geometry of the plant since the aim is to locate each organ within the plant. On the contrary, semantic segmentation assumes that some organ classes (e.g., “stem” and “leaf blade”) have been defined. Prior knowledge about the plant is thus necessary. Both tasks can be solved independently, however, there would be a benefit to solving them simultaneously as prior knowledge about the global plant structure is useful to correct or refine an instance segmentation of the plant. Here, we propose a new approach which alternates between an instance segmentation of the plant based on its local geometry and the use of botanical knowledge to both add semantic labels and detect the defects of this segmentation (see Section 2.1). Our approach uses two graphs that reflect the main scales of analysis: a similarity graph expresses both the local geometry and the neighborhood relationships between the points of the input point cloud, while at a more macroscopic scale, the many points of the similarity graph are clustered into a quotient graph that represents the plant branching structure made up of organs.

A lot of work has already been done on the instance and semantic segmentation of plant 3D point clouds, as we detail below. The proposed approaches are either limited to a couple of organ types (typically, stem vs. leaf blade), require a substantial amount of user interaction (e.g. to create annotated data sets to train machine learning systems), or do not guarantee botanical consistency over the plant structure (e.g. a leaf blade could be directly connected to the main stem). Here, we propose a method that automatically and quickly segments a plant 3D point cloud into labelled instances corresponding to the organs of the plant, with an overall guarantee of the botanical correctness of the segmented result.

### 1.1 Related work

3D point cloud segmentation and classification are problems that have received attention for a long time in the science community.

Early instance segmentation methods can be split into two categories. In the first one, methods compute geometric attributes on the point clouds in order either to grow regions from seed points or to first detect their boundaries and then recover each region from these boundaries. The second one gathers geometric primitive fitting techniques. Our approach fits in the first category. Methods of both types are numerous and surveys can be found in (Nguyen and Le, 2013; Grilli et al., 2017; Xie et al., 2020).

Semantic segmentation can be done directly on the input point cloud according to some pre-defined classes. For this purpose, geometric features are computed locally on each point using its nearest neighbors (Weinmann et al., 2015). Semantic segmentation can also be done on each segment once an instance segmentation has been carried out, by computing geometric features at the segment level and/or using additional knowledge describing how the data is organized at a global level. In both cases, an inaccurate instance segmentation can be corrected or refined, for example by merging neighboring instances with similar features, by splitting an instance, or by interactive operation with the user (Teng et al., 2010). Global knowledge about the shape structure can be expressed as a graph (Landrieu and Simonovsky, 2018; Poux and Billen, 2019) or using an ontology (Hassan et al., 2010; Dietenbeck et al., 2017; Poux and Ponciano, 2020). In our approach, we use graphs to simultaneously segment the plant into its organ instances and give semantic labels to these instances. This allows performing several steps of correction or refinement to recover the best plant structure.

Early automatic and simultaneous instance and semantic segmentation methods for plant 3D point clouds have been based on fitting simple geometric primitives to the plant organs, such as cylinders for stems and branches, combined with implicit prior knowledge about the plant’s global structure. For example (Paproki et al., 2012), start with a coarse segmentation based on growing regions. The main stem is then segmented into its internodes by fitting cylinders. Petioles are also segmented using cylinder fitting. Finally, normal clustering is used to segment each leaf blade into its parts. Similarly (Gélard et al., 2017), first detects the main stem fitting a generalized cylinder. The insertion points of the petioles on the main stem are then detected, which allows to segment each leaf into its petiole and leaf blade. As pointed out by (Ghahremani et al., 2021), such methods lack robustness in the presence of acquisition noise or outliers. As a consequence, the use of a probabilistic framework has recently been proposed to overcome this problem (Ghahremani et al., 2021). The alternative solution to fitting primitives, which we use here, consists of computing local geometric features around each point, using points from its Euclidean neighborhood.

Features should express the local shape of the plant around the point. Two main types of features have been used in the literature. The first one takes the form of a histogram, called the *Point Feature Histogram* (Rusu et al., 2009; Paulus et al., 2013), and allows to segment a plant point cloud into its leaf blades and stems/petioles (Wahabzada et al., 2015). However, additional work is required to segment each organ instance. For example (Vijayarangan et al., 2018), use both a cylinder fitting approach for the stem and region growing for the leaf blades. The second type of features relies on a neighborhood graph built from the point cloud. Features related to a point can be defined either locally as the main directions of the point neighborhood (Elnashef et al., 2019; Wang, 2020), or globally as the set of eigenvectors of a so-called *Laplacian matrix* of this graph (Hétroy-Wheeler et al., 2016; Liu et al., 2018). Instances of the segmentation can be retrieved using the computed features and graph clustering techniques. Each instance can be labelled as either a linear organ (stem, branch, petiole) or a leaf blade. We also use the Laplacian matrix in our approach, but we show that only one of its eigenvectors is enough to accurately segment a plant into its main stem, branches, petioles, leaf blades and apices, as long as the norm and the direction of this eigenvector are processed separately.

Using local features does not ensure any global consistency over the plant structure. To solve this problem, (Vijayarangan et al., 2018) detects intersection points between the main stem and the leaves. (Hétroy-Wheeler et al., 2016; Liu et al., 2018) do not check if the reconstructed architecture is consistent, but only if the desired number of organs is reached at the end of the segmentation process. In the context of forest tree point clouds, (Wang, 2020) organizes instances as nodes of a *superpoint graph*, a concept borrowed from (Landrieu and Simonovsky, 2018), and optimizes this graph to retrieve which instances correspond to wood. Our approach also relies on building a neighborhood graph from the point cloud. As in (Paproki et al., 2012; Gélard et al., 2017; Vijayarangan et al., 2018), we verify that each leaf is connected to the main stem of the plant, but this is done using a quotient graph linking each instance to its neighbors, in a way similar to the superpoint graph of (Landrieu and Simonovsky, 2018; Wang, 2020). This graph allows imposing botanical consistency constraints between organs during the plant structure reconstruction and also allows *a posteriori* control of these botanical rules.

**Figure 1:**
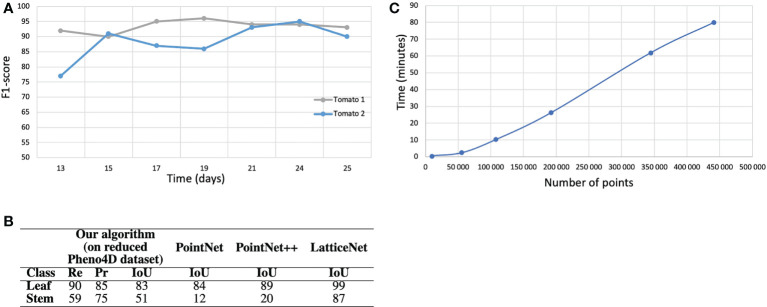
Semantic segmentation results on the tomato plant data set. **(A)** F_1_-score with respect to the stage of development of the tomato plants. **(B)** Semantic segmentation results (in *%* ) on the real tomato plant data set containing two plants at 7 stages of development each. Semantic segmentation *IoU* obtained by (Schunck et al., 2021) with three different neural network on the entire Pheno4D tomato data set. **(C)** Computation time with respect to the size of the point cloud (the four biggest point clouds correspond to the tomato plants).

Machine learning techniques can also be used for simultaneous instance and semantic segmentation of a point cloud. The last years have seen a boom in deep learning-based segmentation methods, as detailed in (Guo et al., 2020). Unfortunately, in the case of plants, most methods only allow two types of organs: stem and leaf blade (Ziamtsov and Navlakha, 2019; Liu et al., 2021; Li et al., 2022a; Li et al., 2022b; Li et al., 2022c), sometimes adding the ground when it has not been removed beforehand (Schunck et al., 2021). Interestingly (Boogaard et al., 2022), proposes a method which distinguishes between stem and petioles, even adding the labels “growing point” (similar to “apex” in our case), “node”, “ovary” and “tendril”. Noticing that leaf blades typically contain more points than the other organs, this method uses the state-of-the-art PointNet++ architecture (Qi et al., 2017) and adds a strategy to counter the effects of such class imbalance. The results of these deep learning-based techniques are generally quantitatively impressive. As a counterpart, they need a large quantity of training data (typically, several hundreds of manually segmented point clouds), which is a very cumbersome manual task. Moreover, the training stage is time and memory consuming and the input point cloud should often be down-sampled to only a few thousand points, which induces a loss of details and potentially of the smaller organs, and restricts for the moment such approaches to small plants. In contrast, our method does not require any training as it uses botanical knowledge within the algorithm. As a consequence, it can handle point clouds with hundreds of thousands of points directly and find a semantic and instance segmentation in a few minutes (see **Figure 1C**).

## 2 Graph-based instance and semantic segmentation method

### 2.1 Method overview

**Figure 2:**
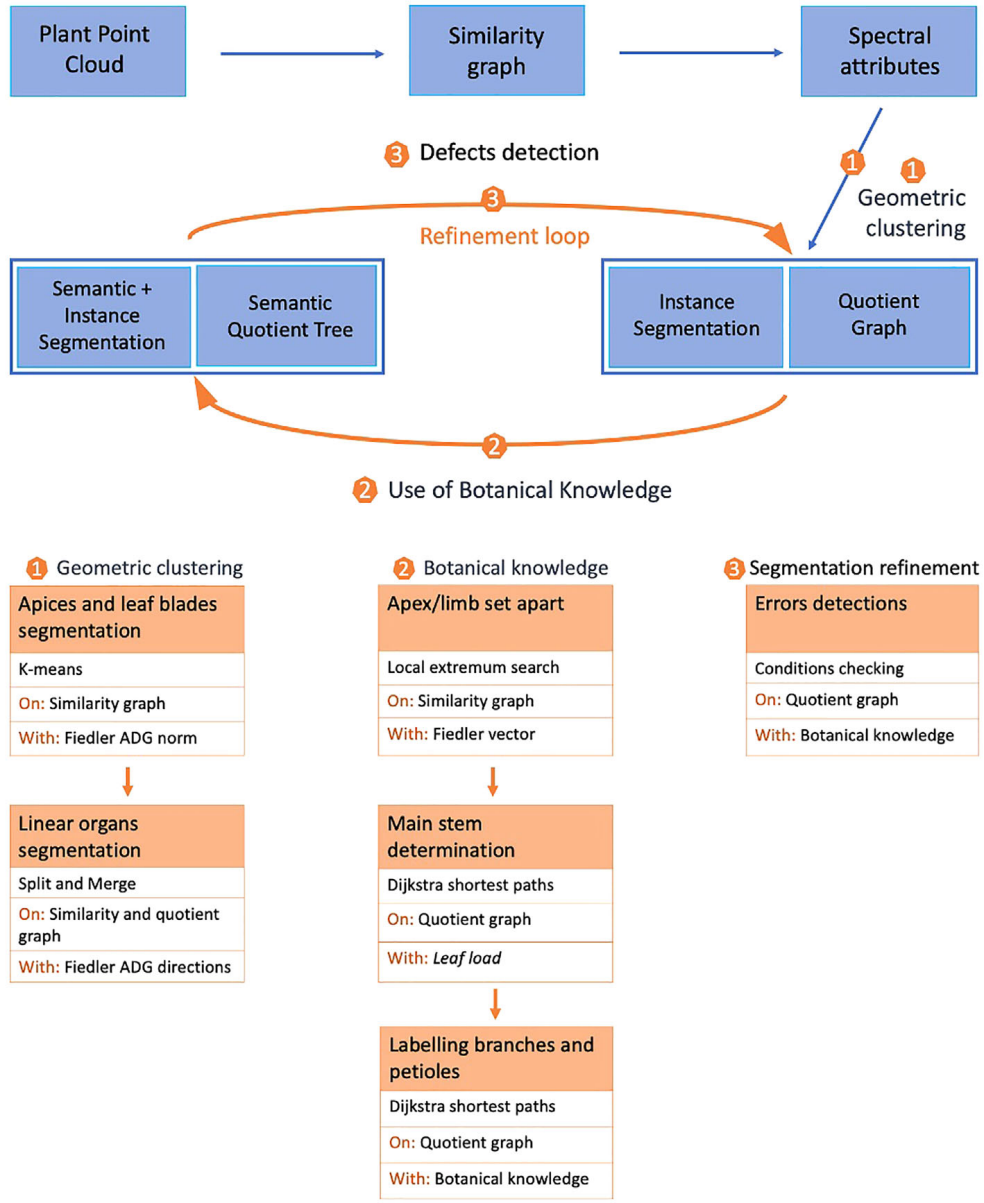
General principle of the mixed instance/semantic segmentation method.

Starting from a 3D point cloud of the plant corresponding to the set of 3D spatial coordinates of each point, our mixed instance/semantic segmentation algorithm proceeds in three main stages, **Figure 2**. In the first stage, we cluster points according to the local geometry of the point cloud. First, a graph is constructed by linking points to their closest neighbors in space (similarity graph). Then, spectral attributes are computed on this graph. These attributes are computed only once as they remain valid during the whole process. This stage, detailed in *Geometric clustering*, produces as an output an instance segmentation of the point cloud and a quotient graph reflecting the macroscopic relationships between adjacent instances (Section 2.2.3).

The second stage of our algorithm aims at modifying this initial clustering using prior botanical knowledge. A label is associated with each cluster, leading to a mixed instance/semantic segmentation. For this, the known architecture of the plant is used in an iterative way to transform the quotient graph into a semantic quotient tree. During this stage, the main stem, branches, petioles, apices and leaf blades are detected, (Section 2.2.3).

Finally, in the last stage, we check if all botanical constraints are met using the semantic quotient tree. If not, the same geometric tools as in the first stage are used locally on the corresponding clusters to refine the segmentation. Once this is done, botanical knowledge can be used again to correct the segmentation in a refinement loop in section 2.4.

### 2.2 Geometric clustering

#### 2.2.1 Computation of spectral attributes: The Fiedler eigenvector

The preliminary steps of our method are meant to extract geometric features from the raw point cloud, in order to get a first clustering of the points. The computed features are also used to correct defects later in the pipeline.

To construct the similarity graph, we first compute the *k* -nearest neighbors of each point. The value of *k* must realize a trade-off between the necessity of keeping a fully connected graph and the need to keep reasonably small the number of edges between nodes to avoid excessive computational times subsequently. We found that a value of *k*=18 generally was making such a compromise.

The similarity graph is thus made of nodes, indexed from 1 to *N* , corresponding to the 3D points and of undirected edges, corresponding to the computed point neighbours. In addition, each edge (*i*,*j*) bears a weight *w*_*ij*_ defining a local measure of similarity between nodes and equals here to the inverse of the Euclidean distance between its two endpoints.

To extract geometrical features from this similarity graph, we used a spectral clustering approach, see e.g (von Luxburg, 2007). The spectral clustering method relies on the Laplacian matrix *L* of the graph. This Laplacian matrix *L* of the similarity graph is defined as:


(1) L=D−A

where *D* is the diagonal matrix of node degrees *d*_*ii*_ (e.g. number of neighbors of node *i* ), and *A* is the graph adjacency matrix, *a*_*ij*_=*w*_*ij*_ if (*i*,*j*) is an edge in *G* and *a*_*ij*_=0 otherwise. Note that *a*_*ii*_=0 for all i=1,..,N. As a result, the elements *L*_*ij*_ of *L* correspond to the degree of node *i* if *i*=*j* , and to the opposite of the weight of edge (*i*,*j*) if *i*≠*j* , and 0 otherwise. By construction, the matrix is of size *N*×*N* with *N* the number of nodes of the graph.

As the Laplacian matrix *L* is symmetric, positive, and semi-definite, its eigenvalues *λ*_*k*_ are real, positive, and correspond to *N* orthogonal real-valued eigenvectors *V*_*k*_ of dimension *N* , such that ∀*k*=1,..,*N* ,


(2) $$LV_{k}=\lambda_{k}V_{k}$$

Each eigenvector *V*_*k*_ consists in a list of scalar values, and each value of this list is associated with a node in the graph. The size of each eigenvector is therefore the size of the graph, *N*. These eigenvectors have a general interpretation on the graph: the eigenvectors corresponding to eigenvalue *λ*_1_=0 identify the different connected components of the graph *G* (here only one as *G* is connected). The next eigenvector, denoted *V*_*F*_ , corresponding to the first non-null lowest eigenvalue, *λ*_1_ separates the graph into two main connected components (corresponding to positive and negative node values in the eigenvector respectively). The following eigenvectors still continue to decompose further the graph *G* into sub-graphs of smaller and smaller sizes (hence the term spectral). These ordered eigenvectors are classically used to cluster 3D point sets using the *K* eigenvectors corresponding to the *K* lowest eigenvalues. However, we found that such a pure spectral clustering method applied to our point clouds was not able to segment satisfactorily plant organs. In addition, the method needs *K* (that would correspond here to the number of plant organs) as an input value, which is most of the time unknown and difficult to estimate *a priori*.

**Figure 3:**
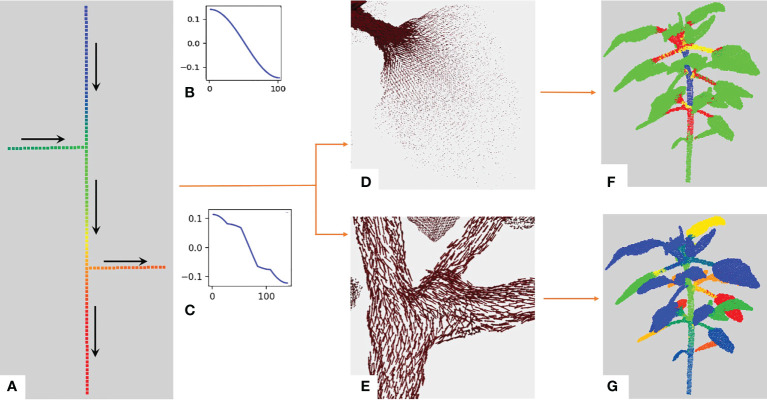
Illustration of the behavior of the Fiedler eigenvector on a toy example. **(A)** Simple example: a linear graph with two side branches. The vertical axis is composed of a hundred nodes chained together. The two remaining branches contain 20 nodes each chained together and connected to a node of the main chain through the first node in their chain. The overall structure makes up a tree of node chains. We computed the Fiedler eigenvector on this graph and its values are shown with a rainbow color scale (red = highest values, blue = lowest values). Arrows indicate the directions of increasing values for the Fiedler eigenvector. **(B)** Values of the Fiedler eigenvector for each node from the bottom to the top of the graph in a) without the two side branches. **(C)** Values of the Fiedler eigenvector on the whole graph in **(A)** with side branches. Slope breaks correspond to extremal nodes or nodes connecting several branches. With the Fiedler eigenvector, we obtain the Fiedler ADG vector field. **(D)** The norm of the vectors drops between the petiole and the leaf blade. **(E)** Unit vectors of the Fiedler ADG vector field are displayed, demonstrating that the directions of the vectors follow the axes of the plant. **(F)** *K* -means clustering obtained using the norm of the Fiedler ADG vector field, with *K*=4 . 2− and 3− dimensional shapes (leaves and apices) are in green while linear shapes are divided into red, yellow and blue. **(G)** Instance segmentation obtained at the end of the first stage of our algorithm. Each instance is in a different color.

The eigenvector corresponding to the first lowest non-null eigenvalue of the Laplacian matrix (eq. 1) is named the Fiedler eigenvector, *V*_*F*_ . During this first analysis, we observed that the variations of the Fiedler eigenvector components over the graph behave in a specific manner in different parts of the plant structure. It has been used to partition a graph into two parts with theoretical guarantees: the number of edges which have one endpoint in each of the two sub-graphs is minimized (Fiedler, 1989; Slininger, 2013). The Fiedler eigenvector basically gives a value to every node according to the longest axis of the graph. In the case of plants, it thus orders the points along the main stem as it is often the plant’s longest organ. This is illustrated on the toy branching system with one main stem and two side branches at different heights depicted in **Figure 3A**. We also noticed an additional interesting property: the variations of the Fiedler eigenvector components show discontinuities at branching points. To see this, let us re-order the nodes of the graph from **Figure 3A** according to their values in the Fiedler eigenvector and plot these values from the highest to the lowest, **Figures 3B, C**. This function shows slope breaks on nodes connecting several side branches of the graph (**Figure 3**), while no such slope breaks are observed in the absence of side branches (**Figure 3**).

To detect such slope breaks in a real, more complex similarity graph, we computed an average directional gradient (ADG) of the Fiedler eigenvector. Let *i* be a node of the graph, *x*_*i*_ be the 3D coordinates of this node and *N*(*i*) the set of neighbouring nodes, *f*(*i*) is the Fiedler eigenvector value at *i* , then the average directional gradient of the Fiedler eigenvector is computed as:


(3) $$\bar{\nabla }f\left(i\right)=\underset{j\in N\left(i\right)}{\sum }(f\left(j\right)-f\left(i\right))\frac{\left(x_{j}-x_{i}\right)}{\left\|x_{j}-x_{i}\right\|}$$

This defines a new vector field on the similarity graph called the Fiedler ADG vector field. Note that we did not normalize by the degree of the node *i* as all nodes in our graph have the same degree (here 18) and that we computed directional gradients on the topological graph without taking into account the edge weights in the similarity graph.

As suggested by the analysis on the above toy example, the directions of the Fiedler ADG vector field overall follow the linear organs of the plant (main stem, branches), and we observe breaks of directions where branching due to the connection of different organs occurs, **Figure 3E**.

Interestingly, we also noticed that the value of the norm of the Fiedler ADG vector field drastically drops between points on a petiole or a branch and points on a leaf blade or an apex, as can be seen in **Figure 3D**. This is due to the fact that petioles and branches are roughly one-dimensional shapes (hence with only one main direction) while leaf blades and apices are mostly two-dimensional and three-dimensional.

Based on these two observations we designed algorithms to cluster the linear organs and to segment flat or volumetric organs of the plant from linear ones.

#### 2.2.2 Apices and leaf blades segmentation

We exploit the norm of the Fiedler ADG vector to automatically detect the apices and leaf blades of the point cloud. As the value of the norm drops on the leaf blades and apices (see **Figure 3D**), we cluster the points with the smallest norm values, using the K-means algorithm. To achieve correct leaf blade and apex segmentations, we observed the segmentation quality varies with *K* . For this, we used an elbow criterion (Satopaa et al., 2011) which made it possible to show that a value *K*=4 was most of the time optimal. Then, the clusters whose centroids have the smallest norm are the clusters containing apices or leaf blades.

This process thus outputs a segmentation of the point cloud in four clusters, with one cluster containing all leaf blades and apices, as illustrated in **Figure 3F** in green. Each of the three other clusters gathers points from the main stem, branches and/or petioles, but each of these organs is not necessarily included in one cluster only. The base of the main stem is also included in this cluster. This is corrected in the following (see Section 2.2.3). Instances of apex or leaf blade can then be retrieved from this cluster with a simple region-growing algorithm on the similarity graph (Vijayarangan et al., 2018). This produces one cluster for each occurrence of apex or leaf blade. However, note that this region-growing algorithm may not produce correct instances if the leaf blades are touching or overlapping. To avoid this issue, a more advanced leaf segmentation process has been proposed in (Li et al., 2019).

#### 2.2.3 Linear organs segmentation

In this stage, we only work with points which have not been detected as part of a leaf blade or an apex (flat or volume shape), i.e. points that belong to the three remaining clusters with linear shape: the main stem, the branches or leaf petioles. Our goal is to cluster points according to their organ class. To do so, we use the direction given by the Fiedler ADG vector field at each point in a “split and merge” approach.

First, to account for the different organ instances in the semantic sub-clusters, each of the three clusters is further divided into connected components. Then each connected component is split into sub-clusters using again the *K* -means algorithm. Here, however, the number of cluster *K* is automatically computed using the classical elbow method with the variance criterion, with a maximum of 20 sub-clusters per cluster (Bengfort et al., 2018). The principle of this algorithm is to compute a range of segmentation solutions by varying the number of clusters. For each solution, it computes a criterion that is plotted as a function of the number of clusters. Then it detects the “elbow” of the curve, the optimal number of clusters after which the criterion does not improve significantly. By splitting the clusters using this method, we often get isolated and small clusters as clustering direction noise enhances the variance value. Besides, the areas where the directions shift are overly segmented.

**Figure 4:**
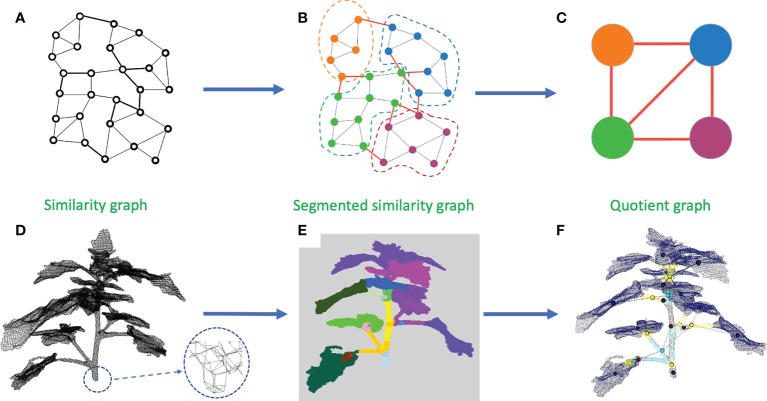
Construction of a quotient graph. **(A–C)** shows the construction pipeline of the quotient graph from the similarity graph. **(A)** Similarity graph. **(B)** Similarity graph with instance segmentation. **(C)** Quotient graph of the similarity graph in **(B)**. **(D–F)** show the same stages on a 3D point cloud. **(D)** Similarity graph with a zoom to see its structure. **(E)** Segmented similarity graph, each color representing an instance of the segmentation. **(F)** Superposition of a segmented point cloud with its associated quotient graph.

A solution to this issue is to merge the clusters depending on their overall directions to avoid taking noise into account. To perform this merging process, we construct a new object. We define an equivalence relation on the points of the similarity graph such that two points are equivalent if and only if they belong to the same computed cluster. Based on this equivalence relation, we construct a *quotient graph Q* by quotienting the similarity graph with this equivalence relation (Godin and Caraglio, 1998). Each (macro-)node of *Q* represents a connected component of a sub-cluster, and edges between nodes represent the adjacency relations between components in the similarity graph (an edge is created in the quotient graph between two (macro-)nodes if at least two (micro-)nodes in these macro-nodes are neighbors in the similarity graph) (Godin and Caraglio, 1998; Sanders and Schulz, 2011). This quotient graph reflects the plant structure at a more macroscopic scale and is much smaller than the original similarity graph (see **Figure 4**). We additionally define a *root node* on the quotient graph. This node corresponds to the bottom part of the main stem. In our plant data sets, it could be defined as the node corresponding to the lowest component of the plant. This automatic criterion is manually checked as in rare cases some branches fall to the ground. In this case, the root node would simply be manually corrected by the user (the case did not occur in our data sets). Nodes corresponding to the leaf blades and apices previously identified are also added to the quotient graph at the end of this stage, with connections to nodes representing linear organs defined according to the similarity graph.

Finally, a high-level merge operation is carried out on the quotient graph to merge potentially over-segmented linear segments. Adjacent nodes of the quotient graph sharing similar directions of the Fiedler ADG vector field are merged in a single node. This is done by computing the mean ${\bar{D}}_{i}$ of the Fiedler ADG vector field direction for each connected component, i.e. for each node *v*_*i*_ of the quotient graph. An energy *E*_*i*,*j*_ is defined on each edge (*v*_*i*_,*v*_*j*_) of the quotient graph, based on the scalar product of the two mean normalized directions of nodes *i* and *j* : $E_{i,j}=1-{\bar{D}}_{i}\cdot {\bar{D}}_{j}$ . Edges with the lowest energy indicate connected segments that have similar directions. They are thus iteratively collapsed and the components represented by their two nodes are merged until a threshold is reached. We experimentally found that a threshold corresponding to an angle of 30^∘^ between ${\bar{D}}_{i}$ and ${\bar{D}}_{j}$ is adequate to stop this merging process. In general, this threshold may depend on the density of the point cloud and/or on the size of the neighbourhood taken to compute the Fiedler ADG vector field.

At the end of this first stage, the initial point cloud is segmented into instances corresponding to either a linear organ (main stem, branch, petiole) or a two-dimensional or three-dimensional organ (leaf blade, apex) of the plant (**Figure 3G**). The quotient graph reflects these instances together with their connections. Note that, at this stage, there is no guarantee these connections are botanically correct, as they are purely based on geometric cues.

The next stage aims at recovering cluster semantics by distinguishing apices, leaf blades, main stem, branches and petioles. This is done using botanical knowledge on the quotient graph to check the plausibility of the connections beween neighboring clusters.

### 2.3 Semantic segmentation using botanical knowledge

From the geometric quotient graph, we then seek to obtain an instance segmentation where each segment is labelled with a unique organ type (apex, leaf blade, petiole, branch or main stem). To reach this goal, prior botanical knowledge is used. We illustrate our approach here on *Chenopodium album*.

We first use knowledge about the shape of the leaf blades to distinguish leaf blades from apices (i.e. bouquet of small and compactly aggregated leaves at the tip of growing axes). Then, we recover the main stem of the plant and use the quotient graph to connect it to the leaf blades and apices. This makes it possible to differentiate branches from petioles.

#### 2.3.1 Differentiation between apices and leaf blades

**Figure 5:**
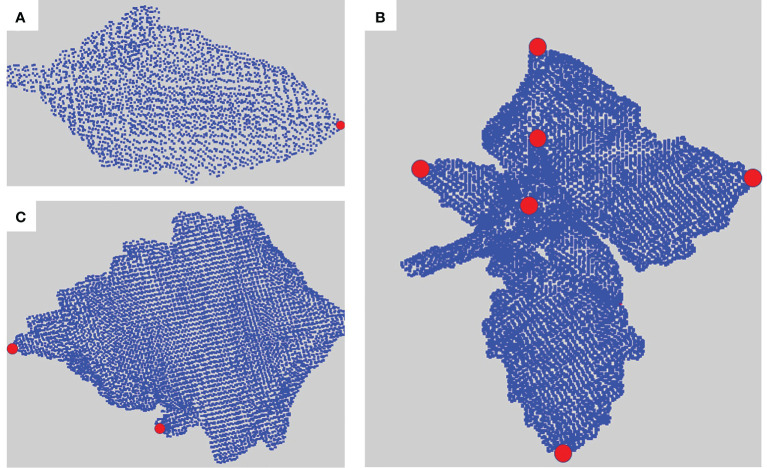
Locally extremal values for the Fiedler eigenvector on three different organs represented by red dots. **(A)** A simple leaf blade, **(B)** an apex and **(C)** a serrated leaf blade.

The Fiedler eigenvector is directly used to separate apices from leaf blades. As shown in **Figure 3A**, a local minimum or maximum value is reached at each end of the point cloud. In each cluster previously classified as either a leaf blade or an apex, we thus look for all locally extremal values of the Fiedler eigenvector on the graph *G.*Extremal values of the Fiedler eigenvector are found using the similarity graph structure. For each node *v* of *G*, we consider the nodes connected to *v* and compare the value *f*(*v*) of the Fiedler eigenvector at *v* with its values at these nodes. If *f*(*v*) is the smallest or biggest value, then we count it as a locally extremal value. Clusters containing only one extremum are classified as leaves (**Figure 5A**) and, otherwise, as apices (see **Figure 5B**). Note that in the case of serrated leaves (see **Figure 5C**), the size of the neighbourhood needs to be increased so that only one local extremum is indeed detected. In the case of multiple-lobed leaves (digitate leaves, for example), a counting system could be set.

#### 2.3.2 Main stem determination

The main stem of the plant is determined by inspecting the quotient graph, starting from the apices and the leaf blades. To do so, we associate a *leaf load* with each node of the quotient graph. The leaf load represents how many leaf blades or apices the organ downstream of the node holds up in the plant. For example, the leaf load is equal to 1 for each apex or leaf blade node, and the load for a branch connected to two leaves should be equal to 2. Note that more elaborate definitions of *leaf loads* are possible, for example using the size (number of points) of the supported apices and leaf blades, or the number of local extremal values of the Fiedler ADG vector field. In our work, we have used the simplest definition described above. Leaf loads are computed iteratively, starting from the apices and leaf blades. At the beginning of the algorithm, we set the leaf load of each node representing an apex or a leaf blade to 1, and the load of any other node to zero.

**Figure 6:**
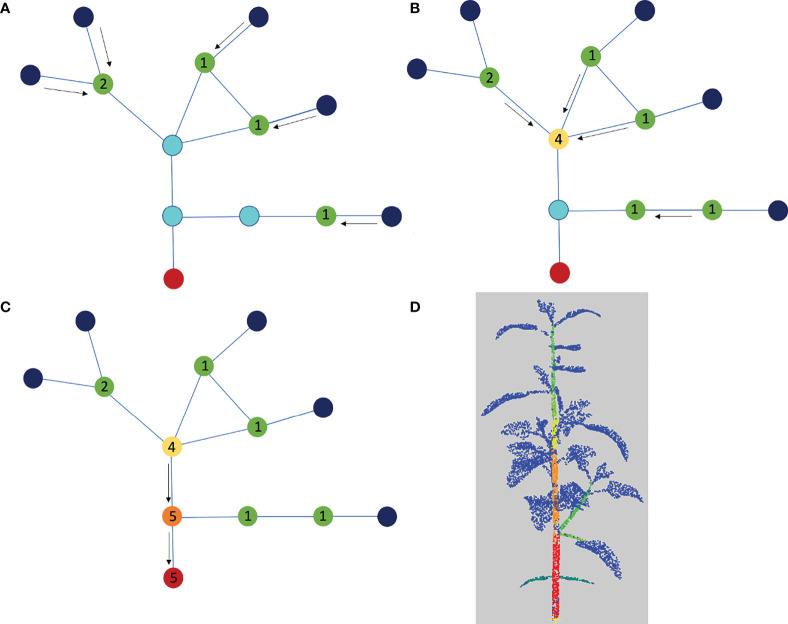
Determination of the main stem using the quotient graph. The root node corresponding to the main stem is shown in red, the nodes representing linear organs are in light blue and the apex or leaf blade nodes are in dark blue. **(A-C)** We compute the shortest paths from each leaf blade or apex node to the root node and add one unit load to each blue node on each path. At the end of the process, the load on each blue node corresponds to the number of shortest paths it belongs to. **(D)** Segmentation color-coded with the loads, from blue (0) to red (3). The blue is associated with the leaf blades and apices.

We then compute for each apex or leaf blade node its shortest path to the root node in the quotient graph. The load of this apex or leaf blade node is added to the load of each other node on this path, see **Figure 6**. Once each path has been computed, we detect the main stem by identifying the path that accumulates the highest load among the shortest paths from apex/leaf blade nodes to the root node. To select the path with the highest load, we actually do not sum all the leaf loads associated with the nodes in the path, as the load of a path would then depend on its number of nodes. Instead, we only count the leaf loads at branching nodes in the graph. For example, on **Figure 6**, the path associated with the lowest leaf blade or apex gives the successive loads 1,1,5,5 along its nodes, and then the total load of the path is computed as 1+5=6 rather than 1+1+5+5=12.

The load on a path only augments at branching junctions in the graph. To keep the algorithm independent of segmentation fluctuations, we count loads only once on a path (i.e. only at branching nodes). For example, on **Figure 6**, the lowest path associated with the lowest limb or apex gives the loads 1,1,5,5 , then the total load of the path is computed as 1+5=6 rather than 1+1+5+5=12. The main stem is identified as the path that accumulates the highest load among the shortest paths from apex nodes to the root node.

All the nodes of the main stem are finally merged on the quotient graph, except the apex node. The corresponding clusters on the point cloud are merged as well.

At this stage, each node of the quotient graph has been labelled as either a *leaf blade*, an *apex*, the *main stem* or a *linear organ*. The nodes labelled as *linear organs* are the last to be processed as they are divided into *branches* and *petioles*.

#### 2.3.3 Labelling branches and petioles

Once the leaf blades, apices and main stem of the plant have been labelled in the point cloud, the remaining points are clustered into branches and petioles. The botanical consistency of the resulting labelled quotient graph is then checked using prior knowledge about the plant species. Prior to this processing, we merge the smallest clusters (typically composed of 10 to 50 points) that may have been produced by the previous stages.

Our goal is thus to transform the quotient graph into a *semantic quotient tree*. We define a *semantic quotient tree* as an acyclic graph derived from a quotient graph in which all edges correspond to plausible connections between organs in a plant. Starting from a quotient graph, we thus need to remove all cycles and check that, for example, a leaf blade is not directly connected to the main stem, or that two leaf blades are not connected to each other. For this, we start by weighting each edge of the quotient graph according to the botanical plausibility of the adjacency of its endpoint labels. This is done by setting a weight equal to 1 on all edges connecting a node representing a linear organ to any other node, and a weight equal to infinity on all the other edges, that is to say, edges connecting two leaf blades, two apices, a leaf blade to the main stem, etc. Edges with infinite weights represent connections which are not botanically possible, that need to be discarded.

From each apex node, we then compute a shortest path to the root node in the weighted quotient graph. If such a path is found with a finite total weight, each node of the path corresponding to a linear organ is labelled as a *branch* node. If not, it means no plausible connection from the apex to the main stem using linear organs can be found in the quotient graph. The connection between the apex and the main stem will remain as an edge but will be detected as an error in Section 2.4.

The same process then applies to the leaf blade nodes. Each node of a shortest path to the root node corresponding to a linear organ is labelled as a *petiole* node if it has not been labelled previously as a branch node and the total weight of the path is finite. If no node is labelled as a petiole, it means the leaf blade node is directly connected either to the root node or to a branch node. Otherwise, the petiole node is necessarily connected to the leaf blade node and either the root node or a branch node, but may also be connected to additional nodes (e.g., to another leaf blade node). In both cases, the inconsistency is detected and the segmentation needs to be corrected, as described in Section 2.4.

At this point, the quotient graph can still contain cycles. Two cases can occur: either a cycle goes through both the root node and a leaf blade or apex node, or not. The first case means that two distinct paths connect the root node to a leaf blade or an apex node in the graph. We tag this leaf blade or apex node for later correction, see Section 2.4. In the second case, unnecessary connections between nodes occur. As some of the edges of the cycle necessarily have infinite weights and were not used in any previous short path, they are simply removed, which eliminates the corresponding cycle.

It may happen that some nodes do not belong to any computed shortest path. They usually correspond to small clusters of points (less than 100 points). They are merged to the adjacent node with which they share the highest number of connections in the similarity graph.

Finally, we give an infinite weight to the edges that have not been used in any shortest path from an apex or a leaf blade node to the root node. In doing so, the sub-graph of the quotient graph containing only edges with unit weights is a botanically plausible semantic quotient tree. The edges with infinite weights of the quotient graph correspond to segmentation errors. They are corrected in the last stage of our method (Section 2.4).

### 2.4 Segmentation refinement

The final stage of our method aims at correcting the remaining botanical inconsistencies in the segmentation. As explained in the previous section, four different types of inconsistencies (corresponding to edges with infinite weights) can remain on the quotient graph.

**Figure 7:**
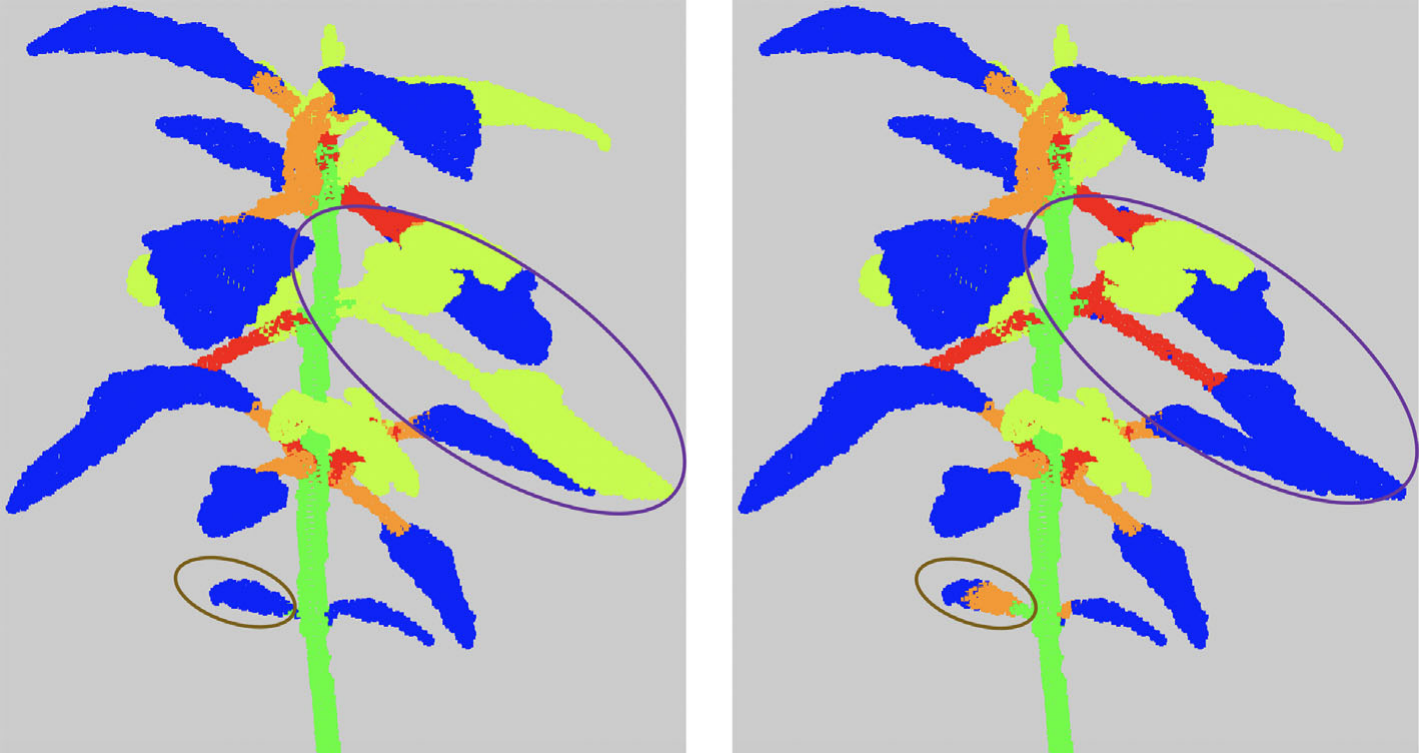
Examples of results of the refinement algorithm, when clusters detected as False are clustered again. The purple circle shows a leaf together with an apex and its branch that were classified in the semantic class *(*Apex). The brown circle shows a cotyledon. As it is not detected differently from the leaf blades, the algorithm considered that the leaf blade of the cotyledon was directly connected to the main stem and therefore clustered it again.

These errors are mostly due to under-segmentations in different places of the plant. In case 1), an apex or leaf blade node is directly connected to the main stem node in the quotient graph. In case 2) a leaf blade node is only connected to a branch node. That means a petiole has not been detected. In both cases, it is suggested that the missing organ has wrongly been clustered with its adjacent apex or leaf blade in previous stages. When a node corresponding to a linear organ is connected to more than two leaf blades or apices nodes (case 3), it is likely the related cluster contains at least two linear organs. Finally, two paths may connect a leaf blade or apex node to the root node (case 4), due to the region-growing algorithm (see Section 2.2.2) that failed on the corresponding leaf blade or apex cluster, which actually contains points belonging to more than one leaf blade or apex, see **Figure 7** for an example.

In all cases, we decide to locally refine the segmentation to solve the problems. The considered clusters are further segmented using the relevant spectral attribute (see Section 2.2): a cluster containing at least an apex or a leaf blade is segmented using the norm of the Fiedler ADG vector field, while a cluster containing several linear organs is segmented using the direction of this Fiedler ADG vector field. The segmentation algorithm used is the *K* -means algorithm, with *K* automatically chosen using the elbow method, as in Section 2.2.3. Note that new clusters are only labelled as *linear* or *two/three-dimensional* organs. This refinement changes the quotient graph, as a node is split into at least two nodes.

In order to retrieve a semantic quotient tree and distinguish between apices, leaf blades, branches and petioles, the semantic segmentation stage (Section 2.3) is repeated. This refinement loop could potentially proceed as long as some defects are found (on our data, we only did one iteration).

## 3 Experimental results

### 3.1 Data sets

We evaluated our method on two plant species with contrasting leaf shapes and architecture, *Chenopodium album* (also known as wild spinach, called Chenopodium in the sequel) and *Solanum lycopersicum* (tomato plant). For the tomato plant, we reused the data set provided by the Pheno4D project (Schunck et al., 2021). For Chenopodium, we created 2 new data sets, one obtained from real plant architecture phenotyping, and the other from simulated computational models of Chenopodium growth. The main characteristics of the different data sets are reported in **Table 1**.

**Table 1:** Main characteristics of the plant data sets used to evaluate our approach.

	Number of point clouds	Number of Ground Truth	Number of Plants used for evaluation	Average number of points	Acquisition	Reference segmentation	Classes available in reference
Chenopodium (real)	22	5	5	10 000	photogrammetry	Instance and semantic	Leaf blade, apex, main stem, branches, petioles
Chenopodium (synthetic)	24	24	24	50 000	simulation	Semantic	leaf (incl. leaf blade and apex), petioles, stem (incl. main stem and branches)
Tomato (real, Pheno4D)	140	77	14	4-5 10^5^	laser	Instance (leafblades only) and semantic	stem (incl. brances, petiole sand and main stem) leaf (incl. leaf blade and apex)

#### 3.1.1 Real Chenopodium data set

**Figure 8:**
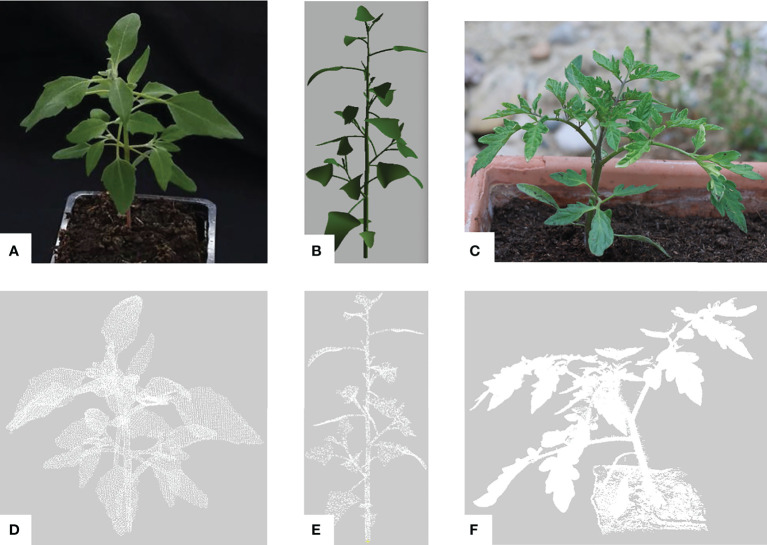
Plant data sets used for the assessment of our pipeline. Pictures of typical architectures from a: **(A)** real Chenopodium, **(B)** synthetic Chenopodium, **(C)** real tomato plant (our photo) illustrating the Pheno4D data set from Schunck et al. (2021). Corresponding raw point clouds processed by our pipeline are respectively shown in **(D-F)**. Point clouds **(D, E)** have the same view angles as pictures **(A, B)**.

Chenopodium is generally considered a weed and has a rapid and monopodial growth, see **Figures 8A–D** for an example. It creates a well-defined main stem that dominates lateral axes that grow out with a slight delay on the leader. The lateral axes in turn then produce lateral axes of their own and so on. The leaves are simple, relatively small and have a triangular shape. The plant growth produces terminal inflorescences after a few weeks only. Here, we considered plants before they reach the inflorescence stage.

##### 3.1.1.1 Plant culture

Chenopodium plants were obtained from commercial seed shops online. Plants were grown in culture chambers on peaty-clay soil (clay: 60*kg*.*m* ^−3^ ), watered with fertilizer (18−10−18*N*−*P*−*K* ) and illuminated with LED lighting (Valoya^©^, sunlight spectrum NS12, 150µ*mol*.*m* ^−2^.*s* ^−1^ ) with day/night regimes of 16h light and 8h dark. Temperature and humidity are controlled as follows: 22°*C* and 60*%* humidity during light, and 18°*C* and 70*%* humidity during nighttime. After a few days of 4°*C* stratifications after sawing, germinated seedlings were transplanted in individual pots at 8 or 10 days after germination (DAG) and plants were imaged between 6 to 12 weeks DAG.

##### 3.1.1.2 Plant imaging

Plants were imaged with 72 pictures taken regularly along a circular path around the plant, using the ROMI Plant Imager (v2, see the online documentation: https://docs.romi-project.eu/plant_imager/build_v2/). This phenotyping robot is equipped with a Sony RX0 RGB camera (1920 x 1060 pixels resolution) attached to a CNC Cartesian arm with one degree of freedom for panning. Optimal imaging contrasts are achieved with a homogeneous black tissue as background and dimmable led lightning. The images were retrieved through the camera’s WiFi interface.

##### 3.1.1.3 Image analysis for 3D point cloud reconstructions

Plant 3D point clouds were reconstructed from the series of 2D images using the method described previously (Wintz et al., 2018). Briefly, it consists of five consecutive steps. First, for each image, the exact position and orientation of the camera, as well as the intrinsics of the camera model (incorporated in the so-called camera matrix) were obtained using a structure-from-motion algorithm [Colmap (Schönberger and Frahm, 2016; Schönberger et al., 2016)]. Second, possible deformations of the images (e.g. created by lens optical aberrations) were corrected using the camera intrinsics computed previously with the OpenCV library (Bradski, 2000). Third, to isolate the plant from other elements in the scene, a binary mask was applied to each image using a linear SVM method where a linear combination S of three red, green and blue channels is computed for each pixel using respective weights of (0,1,0). Final masks are generated using a parametrizable threshold value on S and possible dilation of pixels (computed with the Scikit-image library (Van der Walt et al., 2014), to avoid missing thin elements in the final reconstruction. Fourth, a volume carving algorithm was applied using a regular voxel grid: this outputs a visual hull, which is made by the set of all 3D points whose projection in all (masked) views is inside the reconstructed 3D volume (the size of the projection of each voxel is about the size of a pixel in each of the pictures). Depending on the plant, this step could be restricted to an appropriate bounding box to improve the plant reconstruction and/or computing speed. Fifth, to correct the discretized aspect of the obtained carved surface, we applied a level-set method (Sethian, 1996) to get a smooth estimate of the real surface of the scanned object. The signed distance function needed for this algorithm is computed using a fast marching algorithm implemented in SciPy (Virtanen et al., 2020). The entire code is open source and available online (https://github.com/romi/plant-3d-vision).

The final Chenopodium data set thus contains a total of 22 point clouds, see **Table 1**.

##### 3.1.1.4 Construction of ground truth data

To create a ground truth reference, we selected 5 point clouds in this data set to manually annotate clusters corresponding to the different plant organs. We used the CloudCompare software (CloudCompare, 2022) to separate each organ from the whole point cloud. We could then label it with labels taking into account the organ class and the instance (for example: from 300 to 399 for the apices, from 400 to 499 for the leaf blades). Finally, we merged all the labelled organs together to obtain an instance and semantic ground truth segmentation. We obtain a semantic ground truth segmentation with 5 semantic classes: main stem, branches, petioles, leaf blades, apices.

#### 3.1.2 Synthetic Chenopodium data set

Synthetic plant point clouds (**Figures 8B–E**) were also generated to comparatively evaluate our approach using virtual plants according to the method described in (Chaudhury and Godin, 2020). The development of Chenopodium plants was modelled using L-systems and the L-Py software  (Boudon et al., 2012). The simulated virtual plants were then sampled to obtain ground-truth labelled point clouds. By construction, three semantic classes were available in the models: petiole, stem and leaf blade. This means the ground truth segmentation is a semantic segmentation limited to 3 semantic classes. The model does not contain the apex class. This class was introduced in our segmentation algorithm to represent the aggregated set of small leaves at the extremities of growing stems. An entity with the apex class in the segmentation thus corresponds to a set of terminal aggregated small leaves in the ground truth. We thus considered that apices in the segmented data correspond to the leaf class in the ground truth. Similarly, we merged the branch and main stem classes in the segmented data. 24 synthetic Chenopodium point clouds were generated to evaluate our semantic segmentation. Each point cloud contains 10,000 points. Evaluations were conducted on a 2.6 GHz Intel Core i7 processor and 32 GB of memory. Our segmentation method processes the whole data set of 24 point clouds containing 10 000 points each in an average of 12 minutes in total.

#### 3.1.3 Pheno4D real tomato data set

By contrast with Chenopodium, tomato plants (**Figures 8C–F**) have a sympodial growth and produce composed leaves, generally leading to complex aerial architectures. To test our method on significantly different plant species, we used the tomato plant point clouds of the Pheno4D data set  (Schunck et al., 2021) from which we extracted 14 points clouds corresponding to two different individuals observed at 7 time instants during their growth, **Table 1**. In this data set, the labelling of the points is done according to three classes: soil, stem and leaf (leaf blade), and instances of the leaf blades (but not the stems) are segmented on all point clouds. The reference ground truth for this data set is a semantic segmentation with 2 semantic classes. As for the synthetic Chenopodium point clouds, we have thus merged the apex with the leaf blade classes, as well as the petiole and the branch with the main stem ones. We have also removed the soil in all point clouds as our method does not consider this class. Point clouds of this data set are dense, up to 4 million points without the soil. In order to speed up computation, we down-sampled these point clouds by slightly increasing the minimum distance between any two points. This allowed us to keep 500,000 points maximum per point cloud.

### 3.2 Semantic segmentation evaluation

**Figure 9:**
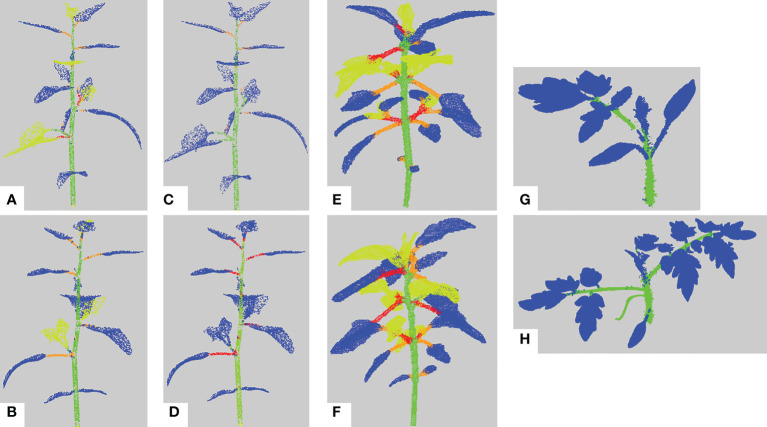
Semantic segmentation results. **(A, B)** Semantic segmentation of two different synthetic Chenopodiums. Points labelled in blue, (resp. green, orange, red, yellow) correspond to leaf blade (resp. stem, petiole, branch, apex) labels. **(C, D)** Same segmentations as in a) and b) where labels of the apex class have been relabeled as leaf blades. **(E, F)** Semantic segmentation of two real Chenopodiums. **(G, H)** Semantic segmentation of two real tomato plants.

We first evaluate the semantic segmentation result of our pipeline on the different data sets. **Figure 9** shows several examples of these results on different plant individuals. In general leaf blades are correctly detected and accurately delineated and the stem is robustly detected. However, petioles are sometimes merged with the corresponding leaf blade or with the stem they are attached to.

To quantify these tendencies, we assessed how well each point has been clustered in the category indicated by an expert (or by the simulation algorithm in the case of virtual plants). For this, we used the following classical metrics used in similar works [e.g (Turgut et al., 2020)].

#### 3.2.1 Evaluation metrics

Let us denote *TP*_*c*_ resp. (*FN*_*c*_ , *FP*_*c*_ ) the number of true positives (resp. false negative, false positive) for the class *c* and *TP* resp. (*FN* , *FP* ) the number of true positives (resp. false negative, false positive) in the whole point cloud. The recall *Re*_*c*_ , precision *Pr*_*c*_ , intersection over union *IoU*_*c*_ and *F*_1_-score are defined respectively as:


(4) $$Re_{c}=\frac{TP_{c}}{TP_{c}+FN_{c}}$$

(5) $$Pr_{c}=\frac{TP_{c}}{TP_{c}+FP_{c}}$$

(6) $$IoU_{c}=\frac{TP_{c}}{TP_{c}+FN_{c}+FP_{c}}$$

(7) $$F_{1}=\frac{TP}{TP+0.5*\left(FP+FN\right)}$$

We compute the mean of all *F*_1_-scores, i.e. a macro *F*_1_-score to obtain an overall score for each data set. Note that the *F*_1_-score is a more relevant measure in our case than the total accuracy (ratio of the number of well-classified points to the total number of points) because of the class imbalance. Since leaf blades contain many more points than petioles, errors in petiole classification would be overlooked by the classification results for the leaf blades.

#### 3.2.2 Results on our data sets

We first tested our approach on our synthetic ground truth data as a control experiment. The pipeline was run on the synthetic Chenopodium data set with and without the refinement stage of our approach (see Section 2.4). This produced segmented point clouds as an output, i.e. points labelled with one of the classes: leaf blade, main stem, branches, petioles and apex. We then applied the above metrics to evaluate these results (remember that to carry out this analysis of the synthetic data set results, we had to merge the main stem and branch classes to a unique ‘stem’ class, and apex and leaf blade to a unique ‘leaf blade’ class), see **Table 2**.

**Table 2:** Semantic segmentation results (in ***%*** ) on the synthetic Chenopodium data set, without and with the refinement stage explained in Section 3.4.

	Without refinement			With refinement		
Class	Re	Pr	IoU	Re	Pr	IoU
Leaf blade	95	92	90	94	94	88
Stem	88	91	81	91	87	80
Petiole	30	68	26	38	51	27

We note that leaf blades were overall well segmented with high recall and precision values and a segmentation score (IoU) of 90*%* . The refinement procedure did not change significantly these scores. These results are consistent with the macro *F*_1_ -scores of 91*%* and 88*%* without and with the refinement, respectively. Similarly, stems are also well segmented with slightly less high scores in general. However, the class petiole has contrasted recall and precision scores, indicating that many truly petiole points were not recognized as part of a petiole, but that if points were recognized as petiole, they were indeed truly petiole points. IoU confirms that a majority of truly petiole points were not correctly identified as petioles by the algorithm (about 74%).

**Figure 10:**
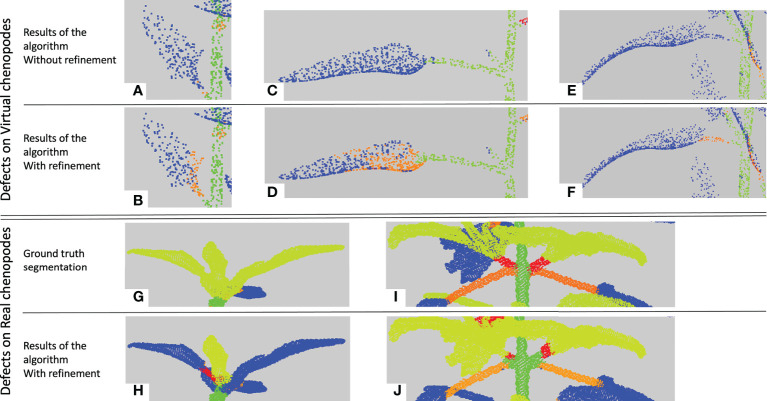
Main segmentation errors and refinement corrections. Five examples of semantic segmentations of different plant parts **(A-J)**. **(A-F)** are presented without (top row, a, c, e) and with (bottom row, b, d, f) refinement. **(G, I)** are the ground truth labelled point clouds of the part segmented by our algorithm in **(H, J)**. Points labelled as on a petiole are in orange, on a branch in red, on a leaf blade in blue, on an apex in yellow-green and on a stem in green.

The low figures for the petiole class can be explained by three main factors. First, petioles are usually small organs and therefore contain much fewer points than stems and leaf blades. As the boundaries between organs are not precisely determined both by manual and automatic segmentations, there may be greater point classification errors close to organ borders. In the case of petioles, these arbitrary errors at both extremities of such small organs can significantly impact the overall segmentation quality of the organ (see **Figure 10A**), despite the fact that the organ is actually recognized (**Figure 10B**) as reflected by the limited precision score in **Table 2**. Second, if the algorithm wrongly detects a leaf as an apex, then the petiole will tend to be wrongly interpreted as a stem, thus leading to a wrong classification of all its points. Finally, the petiole might be merged by the algorithm with its leaf blade, also leading to a wrong classification of all its points (**Figure 10E**).

Contrarily to the case of real plants (see below), the refinement procedure does not improve the results for the petiole class in the case of synthetic plants. This can be explained in several ways. First, if the petiole has initially been included in a stem, the refinement stage looks for a petiole in the leaf blade point cloud rather than in the stem, degrading the result for the leaf blade cluster instead of improving that of the stem (**Figures 10C, D**). Second, when the petiole is merged with its leaf blade, this cluster is often labelled as an apex and the refinement will correct it to an apex plus a branch rather than a petiole. Third, if the corresponding leaf blade has been correctly delineated but labelled as an apex, the fact that the petiole has been labelled as a branch is not considered a defect by the algorithm that thus does not correct it. The refinement however improves the results, as shown in **Figures 10E, F**), when the petiole has been labelled as part of a leaf blade.

Altogether, for synthetic plants, the refinement procedure did not have a good balance between correcting errors and producing new ones. However, we will see that this procedure may have a positive impact on the segmentation of different organs for real plants.

We then tested our algorithm on five Chenopodiums from the real data set for which a ground truth segmentation was manually made. Here, this manual ground truth made it possible to reuse the exact same classes as the ones produced by the algorithms. We, therefore, did not have to merge any of the original classes as we did in the case of synthetic plants. Quantitative results are given in **Table 3**.

**Table 3:** Semantic segmentation results (in *%* ) on the data set of 5 real Chenopodiums, without and with the refinement stage explained in Section 2.4.

	Without refinement			With refinement		
Class	Re	Pr	IoU	Re	Pr	IoU
Leaf blade	85	76	70	90	79	73
Main stem	90	78	73	94	76	73
Apex	72	90	69	73	95	71
Petiole	52	38	26	54	39	29
Branch	52	18	16	58	18	16

In general, results are comparable to the ones obtained on the synthetic plant data set, if we consider classes that appear in both tables (note that the ‘Stem’ class from the previous table is split here into ‘Main stem’ and ‘Branches’). The macro *F*_1_ -score is 78*%* and 80*%* without and with the refinement, respectively. Quantitative scores are however slightly lower for leaf blades and stems but are now mostly substantially improved by the use of the refinement procedure. Contrary to synthetic plants, recall scores are now markedly higher than precision, meaning that the algorithm identified in these classes a larger number of points that do not belong to them, probably due to greater variability of the organs' shapes. Interestingly, the new ‘Apex’ class, figuring the terminal bouquet of small leaves at the end of plant axes, has on the opposite a high precision score while keeping a limited recall value. This means that points detected as apices were correctly classified but that some apex points were missed and detected as part of another organ (**Figures 10G, H**). Petioles and branches have similar scores that together reflect the similar scores obtained in the synthetic plants’ experiment. Note that the branch class contains fewer points than the petiole class in the real Chenopodium data set, see **Figures 10I, J**.

Finally, to test the robustness and ability of our pipeline to handle other plant species, we evaluated it on a data set of 14 tomato plant point clouds. We excluded seedlings as our method applies to already developed branching structures. As only two semantic classes, ‘Leaf’ and ‘Stem’, were available in this data set, we merged our classes ‘Apex’ and ‘Leaf blade’ into a ‘Leaf’ class, and ‘Main stem’, ‘Petiole’ and ‘Branch’ into a single ‘Stem’ class. Results are shown in **Figure 1B**. We did not adapt our refinement algorithm to the case of tomato plants and therefore discarded the refinement stage in this case.

Despite a markedly different overall plant architecture compared to Chenopodium, the algorithm was able to correctly segment a large majority of the points (F1-Score: 85 *%*). As in the above experiments for real and synthetic Chenopodiums, leaf segmentation scores were high. Stems scores were lower. However, as points could be labelled with either one of the ‘Leaf’ or ‘Stem’ classes, one can see that the quantity of points that were wrongly classified by the algorithm in one class contributes exactly to the errors for the other class. For example, a recall of 59 *%* for stems means that 41 *%* of the stem points were badly classified as leaves. These 41 *%* of points actually make up the 15 *%* of failed points indicated in the Leaf precision score. Hence, the difference in intensity between these scores reflects in part the relative quantity of points in the different classes. These results can be compared to those obtained in (Schunck et al., 2021) where the authors used the whole set of tomato plants available and segmented the data set with three different deep neural networks: PointNet (Qi et al., 2017), PointNet++ (Qi et al., 2017) and LatticeNet (Rosu et al., 2019). The *IoU* for the classes leaf and stem were respectively 84 *%* and 12 *%* for PointNet, 89 *%* and 20 *%* for PointNet++ and 99 *%* and 87 *%* for LatticeNet. While our algorithm performs better than the PointNet and PointNet++ networks for stem detection, it is not as accurate as LatticeNet on this dataset.

We also checked how these scores depended on the stage of development of a tomato plant (**Figure 1A**). The 14 individuals in the tomato plant data set actually correspond to 2 plants observed at 7 stages of development. The curves suggest that the segmentation results do not depend on the stage of plant development, at least as long as the plant architecture complexity remains reasonable.

### 3.3 Instance segmentation evaluation

We used the real Chenopodium data set to perform an evaluation of our instance segmentation method, and a set of statistical indicators commonly used to evaluate instance segmentation of point clouds, e.g. in (Chen et al., 2021). The Rand index defines a similarity measure between two segmentations, e.g. our automatic instance segmentation and the corresponding ground truth, by looking at the proportion of pairs of points that are rightly or wrongly appearing in the same or in different clusters (Pedregosa et al., 2011). The adjusted Rand index corrects this raw index to obtain a value between 0 and 1, 0 corresponding to a random segmentation and 1 to an exact matching between clusters. In the case of size disparities between the clusters (e.g. between petioles and leaf classes), an (adjusted) mutual information score provides an adequate metric to compare segmentations (Romano et al., 2016). Here again, a value of 1 means that the two segmentations are identical, while two segmentations with independent labels will have a score around 0 (it can be negative).

We also use two complementary indices: completeness and homogeneity, that reflect the quality of individual segmented clusters (Rosenberg and Hirschberg, 2007). Completeness measures how much a cluster contains all of the points that should be in it and if it missed a lot of points, while homogeneity measures how much each cluster only contains points of a single class (Rosenberg and Hirschberg, 2007).

Results reported in **Table 4** show that if class size disparity is properly corrected for (here by the Adjusted mutual information score), the quality of our instance segmentation is quite correct (index = 0.89) indicating that a large majority of points that should be found in the same cluster is indeed in the same cluster. The high homogeneity score means that clusters made by the algorithms correspond mainly to a unique cluster of the ground truth segmentation (but which may occasionally be badly labelled). The relatively high completeness score indicates that around 85% of the points on average in each instance cluster are correctly found in the same cluster.

**Table 4:** Mean scores to evaluate instance segmentation results on the real Chenopodium data set, without and with the refinement stage explained in Section 2.4.

Score	Without refinement	With refinement
Rand	0.96	0.97
Rand adjusted	0.79	0.82
Adjusted mutual information	0.89	0.91
Completeness	0.85	0.87
Homogeneity	0.94	0.95

For the five plants, on the total 28 leaf blade segmented instances obtained with the refinement procedure, we computed a mean recall of 97*%* , a mean precision of 99*%* and a mIoU of 97*%*, showing that the leaf blades points were faithfully identified by the algorithm.

### 3.4 Extraction of phenotypic traits with agronomical value

Finally, we aimed to assess whether the method was able to faithfully extract phenotypic traits classically used in agronomical applications. Among others such as plant volume, leaf area density, surface-to-area ratio, normalized difference vegetation index, etc., we selected stem height and leaf area index (LAI) as they are derived from reconstructed stems and leaves respectively. Both traits characterize the plant growth status. Stem height is directly linked with the plant’s primary growth and its variation in time is a key characteristic of the plant dynamics and response to the environment. LAI is usually defined for crops and corresponds to the amount of (one-sided) leaf area above one unit surface of ground. It is a dimensionless quantity that may range between 0 (non-covered soil) to several units in dense canopies. This index is physiologically significant as it integrates various aspects of crop physiological status: the height of the plant, the size of the leaves in different height strata, and their orientation in space. It is commonly used in ecophysiological models as a key parameter to estimate direct light interception by crops (through the Beer-Lambert law), and thus a key variable to build estimates of plant photosynthesis.

We tested the ability of our method to extract these high-level phenotypic traits on the five chenopodiums for which ground truth was available. The height of the plant was estimated as the main stem length extracted from our segmentation algorithm and compared to ground truth data (**Table 5**).

**Table 5:** Comparison of total leaf blade areas and LAI values between the ground truth and the segmentation performed on 5 real Chenopodiums, as well as comparison of the main stem height computed on 5 synthetic Chenopodiums on ground truth and segmented models.

		Total leaf blade area (mm²)		LAI		Main stem height (mm)	
Plant	Bounding box area (mm^2^)	Ground truth	Segmented	Ground truth	Segmented	Ground Truth	Segmented
Chenopodium 1	6397,1	1216,9	1172,6	0,19	0,18	70,3	71,0
Chenopodium 2	5117,6	1420,2	1395,4	0,28	0,27	71,8	74,9
Chenopodium 3	4915,8	1745,4	1734,9	0,36	0,35	41,4	44,1
Chenopodium 4	8655,9	4060,7	4008,1	0,47	0,46	62,7	63,5
Chenopodium 5	6254,1	1895,7	1887,3	0,30	0,30	70,3	69,0

The average error on the plant height was less than 2,3 *%* and suggests that the method could work on larger data sets when larger ground truth data will be available. To estimate LAIs, we first computed a Bézier surface (Boukhana et al., 2022) on each leaf blade defined by the ground truth segmentation. We did the same for the leaf blades defined by the segmentation obtained by our algorithm and compared the obtained surfaces, see also **Table 5**. The 28 resulting leaf blades had an average surface of 377 *mm* ^2^ with a standard deviation of 7,85 *mm* ^2^ . We then estimated the LAI by computing the bounding box of each plant and taking the surface area of the bounding box basis as a proxy for the ground surface above which leaf surface area was computed.

These first results of our phenotyping pipeline at an integrated level show that physiologically meaningful canopy parameters can be extracted with a fairly good accuracy (mean relative error of 2,3 *%* and 3,7 *%* standard deviation for plant height and -1,5 *%* and standard deviation of 2,3 *%* for leaf blade). The analysis of more plants will help confirm this trend when additional ground truth data will be available.

## 4 Discussion

The automatic and simultaneous semantic and instance segmentation of 3D plant models is an important issue for automatic plant phenotyping. Recent approaches are restricted to specific species and/or to very few classes (typically, stem vs. leaf). A recent trend has been to use deep neural networks and is showing promising results. However, these approaches suffer from inherent limitations as i) point clouds usually need to be drastically down-sampled, hence loosing details such as petioles, flowers, etc., ii) a massive quantity of annotated data is required to train the network, iii) no guarantee is given over the global botanical consistency of the segmentation, e.g., a leaf blade could be directly connected to the main stem, or leaves connected to other leaves.

Our work demonstrates that geometry-based methods can still be efficient while not (or less) suffering from these drawbacks. By combining robust local geometric features and global botanical knowledge encoded in our graphs and algorithms, our approach generates in a few minutes segmentations semantically correct and whose instances are accurately delineated, for point clouds containing up to hundreds of thousands of points. We have shown that the norm of the average directional gradient of the Fiedler eigenvector is efficient to segment two-dimensional organs such as leaf blades and apices, while its direction can be used to identify linear organs. A quotient graph connecting each organ to its neighbors is a useful tool to optimize this classification using macroscopic semantic information and check botanical consistency over the branching structure of the plant.

Our preliminary results show that organs are accurately demarcated. In particular, the norm of the Fiedler ADG vector field is efficient in differentiating two-dimensional and linear organs, as expressed by the recall, precision and IoU scores for the leaf blade class in **Table 2**. This supports the idea of using spectral components such as the Fiedler eigenvector, in place of, or complementary to, the classical geometric tools (linearity, planarity, scattering) used on human-made shapes (see e.g (Landrieu and Simonovsky, 2018). Our experiments on Chenopodiums and tomato plants tend to show that the Fiedler eigenvector could be equally used for various plant species. Our approach is fast, computationally cheap and does not require extensive parameter tuning, as we always use *k*=18 neighbours for the construction of the similarity graph and *K*=4 for the apices and leaf blades *K* -means segmentation. Our experiments also showed that the results are at least as good as non-specialized neural networks such as PointNet and PointNet++.

Spectral clustering approaches are known to be robust to non-uniform point density and acquisition noise. However, in our case, special attention must be taken to the computation of the average directional gradient of the Fiedler eigenvector (Eq. 3). For simplicity’s sake we currently use the *k* nearest neighbours at each point; using the neighbours in a sphere centred at the point would be more robust in the case of non-uniform sampling but would require adapting our definition.

Using only geometry-based features is not enough to accurately segment a point cloud, as it is for example hard to discriminate petioles from branches (Boogaard et al., 2022). Our proposal to include botanical knowledge encoded as a semantic quotient tree into the segmentation process enables us to control the consistency of the segmentation and refine it if necessary, both semantically (e.g., deciding between petioles and branches) and per instance (e.g., to split a petiole from the associated leaf blade). Such a semantic quotient tree is easy to define, compute and modify. Furthermore, it will make it possible to extend the evaluation of our algorithms at a macroscopic scale by comparing the output tree structures from instance segmentation with the ground truth ones at a global level. For this more work needs to be done to use tree comparison metrics (Ferraro and Godin, 2000; Boudon et al., 2014) on quotient trees to assess results at more macroscopic levels.

Preliminary results show that our approach reaches competitive quantitative results in terms of classical classification metrics (precision, recall, *F*_1_-score), especially for the leaf blades. However, we believe these results can still be improved using more advanced clustering techniques than the *K* -means algorithm. We have provided an example of how botanical knowledge could be encoded to define a semantic quotient tree. Other rules or semantic classes could be added and a full method could be developed to express and integrate botanical knowledge in our algorithms in a more systematic way. In the tomato plant case, for instance, rules could be defined to isolate composed leaves (made of several leaf blades and stem segments) for instance or to integrate fruits and flowers in the segmentations in a botanically consistent way.

## Data availability statement

The datasets presented in this study can be found in online repositories. The names of the repository/repositories and accession number(s) can be found below: https://zenodo.org/record/6962994#.YuvYkS8itqs.

## Author contributions

KM, CG, and FH-W conceived and designed the study, designed the algorithms, analyzed the results and wrote the paper (with inputs from other authors). KM implemented the algorithms, run the pipeline on the different data sets and produced the figures. CG developed the synthetic model of Chenopodium. JC and FB designed the real Chenopodium phenotyping experiment and produced the Chenopodium 3D point cloud data set. MT and KM annotated the real Chenopodium data set to produce the ground truth data. All authors read and approved the final version of the manuscript before submission.

## Funding

This work has received funding from the European Union’s Horizon 2020 research and innovation program under grant agreement No 773875 (EU-H2020 ROMI, Robotics for Microfarms).

## Acknowledgments

The authors would like to thank Guillaume Cerutti, Frédéric Larue and Joris Ravaglia for fruitful advice on parts of the pipeline implementation.

## Conflict of interest

The authors declare that the research was conducted in the absence of any commercial or financial relationships that could be construed as a potential conflict of interest.

## Publisher’s note

All claims expressed in this article are solely those of the authors and do not necessarily represent those of their affiliated organizations, or those of the publisher, the editors and the reviewers. Any product that may be evaluated in this article, or claim that may be made by its manufacturer, is not guaranteed or endorsed by the publisher.
